# Biomass formation and yield performance in diverse multicrops and their potential for biofuel use in short-growing boreal climate conditions

**DOI:** 10.1038/s41598-026-46324-0

**Published:** 2026-03-30

**Authors:** Jovita Balandaitė, Kęstutis Romaneckas, Rasa Kimbirauskienė, Aušra Sinkevičienė, Aušra Marcinkevičienė

**Affiliations:** 1 Department of Agroecosystems and Soil Sciences, Agriculture Academy, Vytautas Magnus University, Kaunas Reg., Lithuania; 2 Bioeconomy Research Institute, Vytautas Magnus University, Kaunas Reg., Lithuania

**Keywords:** Biomass, Biofuel pellets, Environment, Faba bean, Industrial hemp, Maize, Multicrops, Ecology, Ecology, Environmental sciences, Plant sciences

## Abstract

Herbaceous plant biomass is an important resource for energy production and an effective strategy to ensure energy supply independence. Growing agricultural plant mixtures (multicrops) contributes to increasing biomass yields, enhancing farm biodiversity, improving soil health, and promoting environmental sustainability. However, most crop mixtures have not been studied at all. For this reason, stationary field experiment was conducted from 2020 to 2022 at Vytautas Magnus University, Agriculture Academy, Kaunas reg., Lithuania. The aim of the study was to evaluate the development and productivity of maize, industrial hemp and faba bean single crops and its binary and ternary mixtures, as well as the related energy and environmental aspects of the applied agrotechnologies, under short growing season conditions. Faba bean ecosystem services improved maize and industrial hemp growth in mixtures, increasing plant height by 14%, leaf assimilation area by 24%, and chlorophyll index by 19%, indicating enhanced nitrogen availability and photosynthetic activity in companion crops. Although biomass of individual species decreased in more diverse mixtures, total plot biomass increased, with the ternary maize–hemp–faba bean crop producing 4–8 times more dried biomass than single crops in the first year. The ternary system achieved the highest Comprehensive evaluation index (CEI = 4.54) and the greatest net energy yield (367,668 MJ ha⁻^1^) despite higher fuel consumption (103.3 L ha⁻^1^). Pellets produced from ternary biomass met quality standards (density 1238 kg m⁻^3^, ash 6%, ash shrinkage temperature 1042 °C), while Life cycle assessment showed lower environmental impacts for mixed-crop biomass compared with single maize crop (29.1 kg CO₂ eq GJ⁻^1^). Overall, diversified cropping systems demonstrate strong potential for sustainable biomass production and environmentally efficient bioenergy generation.

**Supplementary Information:**

The online version contains supplementary material available at 10.1038/s41598-026-46324-0.

## Introduction

In recent years, the negative impacts of climate change have intensified. The warming climate exacerbates soil degradation due to intense rainfall and drought periods, reduces biodiversity and crop productivity potential (especially in southern countries), and negatively impacts economic returns^1^. In Northern countries, crop productivity potential increases due to the prolongation of the vegetative season. For example, in Lithuania, the vegetative season of crops has been extended by at least 2**–**3 weeks over the last 15 years. This allows for larger quantities of agricultural production, especially biomass. This potential is not yet fully exploited, especially in biofuel production systems, though plant biomass is one of the most important sources of renewable energy^2,3^. For now, the focus is on growing woody plant biomass and using forest waste.

The use of plant-based biofuels significantly reduces the greenhouse effect: CO_2_ emissions are close to zero, as the CO_2_ released during combustion is used to produce organic matter during photosynthesis^4^. The combustion of biofuels reduces emissions of sulfur compounds to the environment, and the ashes produced can be used to fertilize the soil^5^. The development of biofuel or another environmentally friendly component production from plant biomass would contribute to the sustainability of agroecosystems, which is aimed at the circular economy^6–8^.

Growing monocropped cultivations are still widespread in most countries to increase farm income, regardless of their negative environmental impacts^9^. Multicropped cultivations provide the opportunity to obtain a more varied multiple harvest during the vegetative season. It also has a positive effect on ecosystem services, helps to maintain and restore ecological balance through biodiversity, increases productivity and income, requires fewer inputs, reduces emissions, and increases the crop’s resilience to climatic shocks by increasing the C content of the soil^10,11^. Growing multicrops helps prevent the spread of weeds, diseases, and pests^12^. Growing crops together stimulates the activity of soil microorganisms, which accelerates humification processes^13^. The roots of multicrops are in different soil layers, allowing them to take up nutrients and water from different locations and thus supply them more efficiently. Growing together, plants of different species position their leaves at distinct height layers and spatial arrangements, enabling more efficient light capture, reduced mutual shading, and enhanced photosynthetic performance^14^. Multicropping is also a promising strategy to increase land-use efficiency and cost-effectiveness of farming, especially for resource-constrained countries^15^.

Industrial hemp (*Cannabis sativa* L.) is a fast-growing, high-fiber crop increasingly recognized as a promising renewable feedstock for bioenergy and biobased materials. Depending on environmental conditions, cultivar, and soil fertility, hemp can produce approximately 5.5–8.5 t ha⁻^1^ of dry biomass, making it a competitive lignocellulosic energy crop^16,17^. The crop requires relatively low agricultural inputs, suppresses weeds through rapid canopy closure, and improves soil structure through its extensive root system, which makes it suitable for crop rotations and cultivation on marginal lands^18–20^. Hemp is cultivated globally—particularly in China, Canada, France, and the United States—and generally performs best in well-drained soils under temperate to continental climatic conditions, with optimal temperatures of approximately 16–27 °C and seasonal precipitation of 400–800 mm, although cultivar-specific tolerance to environmental stresses varies^21–24^. In addition to agronomic advantages, hemp stems possess favorable fuel characteristics, including high volatile matter and calorific value and relatively low ash, nitrogen, and sulfur contents Consequently, hemp biomass and processing residues are increasingly considered suitable feedstocks for thermochemical and biochemical conversion pathways, enabling the production of bioethanol, biodiesel, biohydrogen, organic acids, and other value-added biomaterials within integrated biorefinery systems^25,26^. In Lithuania, 2 500 ha of hemp have been declared for 2023^27^. Hemp is grown mainly for seed production, to a lesser extent for fiber for the textile and construction sectors. The need for hemp for biofuel production is undeveloped.

Maize (*Zea mays* L.) is a globally important cereal crop with significant potential as a biomass and bioenergy feedstock in addition to its traditional roles in food and feed. Measured above‑ground biomass yields in improved maize hybrids typically range around ~ 18–22 t ha⁻^1^ under good agronomic management in temperate environments, providing substantial material for biofuel and lignocellulosic bioproduct conversion. Maize stover—the stalks, leaves, husks, and cobs left after grain harvest—is one of the most abundant lignocellulosic biomass resources worldwide, comprising roughly 47–50% of total plant dry mass, and has significant potential for renewable energy and bioproducts^28^. Valorization of maize stover for biogas and biofuel production can improve energy output and system efficiency while reducing reliance on fossil fuels^29^. Breeding dual-purpose cultivars allows for simultaneous improvement of grain yield and stover quality for bioenergy conversion, enhancing the crop’s overall biomass potential^30^. Sustainable residue management is essential to maintain soil health and prevent erosion, ensuring long-term productivity and environmental sustainability^31^. In In Lithuania, 52 400 hectares of maize have been declared as lacking in 2023^27^. 70% of the maize is grown for biomass feed production, 30%—for grain. The cultivation of maize biomass for the energy sector is underdeveloped.

Faba bean (*Vicia faba* L.) is a cool‑season grain legume widely cultivated for its high protein seeds and efficient symbiotic nitrogen fixation, which can supply much of the plant’s nitrogen demand and reduce synthetic fertilizer use^32^. Compatible rhizobium strains enhance nodulation, shoot and root biomass, and overall yield, demonstrating the importance of microbial interactions in sustainable production systems^33^. Field studies confirm that inoculation with nitrogen‑fixing bacteria improves both seed yield and protein content, emphasizing the role of faba bean in environmentally friendly cropping systems^34^. Faba bean is a sustainable crop that not only provides ecological services but also leaves a large amount of residual biomass after the grain harvest, which can be used for energy production^35^. In 2023, 82 000 hectares of faba beans were grown in Lithuania^27^. Faba beans are grown only for seed production, but their residue biomass is suitable for producing dry biofuel^36^. In addition, in Lithuania, the dried biomass residue capacity reaches 4**–**5 t ha^−1^.

The above mentioned plants could also be mixed as multifunctional crop, as industrial hemp warrants high biomass yields and protection from pests and some diseases with low competition for sunlight. Maize ensures high crop productivity, and it has few diseases and pests in the Nordic countries. Faba beans provide ecological services to the cultivation by naturally providing companions with not only nitrogen but also potassium.

The novelty and actuality of our research are: 1) ternary hemp, maize, and faba bean multifunctional crops for effective growth of abundant biomass have not yet been studied in general. That is the main novelty of study. The characteristics of their development (primarily as continuing cultivation for 3 years) in mixtures were not known; 2) the pesticide-free approach was used, which is not usual in modern-intensive agriculture; 3) crops were grown for a short time, 103–105 days after germination, until the faba beans riche final maturity. This ensures an early supply of plant biomass for fuel production without loss of overall productivity. Other non-legumes companions can grow for longer and provide a later biomass harvest for processing; 4) the energy and environmental balance of multicropping technologies was assessed, and a Life Cycle Assessment (LCA) of the produced fuel pellets was performed, which were not been analyzed before; 5) the technological parameters for producing fuel pellets from a ternary crop were registered in the Patent Office of the Republic of Lithuania as an invention^36^.

The aim of the present study was to evaluate how diversified short-growing cropping systems (binary and ternary mixtures of maize, industrial hemp, and faba bean) affect: a) biomass productivity, plant physiological traits, energy balance, pellet quality, and life‑cycle environmental impacts compared with monoculture (single crop) production, b) to identify the cropping configuration that maximizes net energy yield and minimizes environmental burdens for sustainable bioenergy production. We hypothesize that in boreal climate conditions, short-grown diversified crop mixtures produce higher plot-level biomass, greater net energy, equal or better fuel pellet quality, and lower LCA impacts than monocultures.

## Materials and methods

### Site description

In 2020–2022, single, binary, and ternary crops of maize (*Zea mays* L.), industrial hemp (*Cannabis sativa* L.), and faba bean (*Vicia faba* L.) were sown at the Experimental Station of Vytautas Magnus University Agriculture Academy (VMU AA) at the following coordinates: 54°53′7.5″ N 23°50′18.11″ E. The Experimental Station is located on the south-western side of Kaunas city, on the left bank of the Nemunas River, in the central part of Lithuania (Supplementary Fig. 1).

The soil at the experimental site is a silty loam (46% sand, 42% silt, 12% clay) Endohypogleyic-Eutric Planosol (Ple-gln-w)^37^, with a texture of silty light loam on heavy loam. A soil pH_KC_ was 7.3–7.8, total nitrogen content—0.08–0.13%, organic matter content—2.6–2.9%, available potassium—118 mg·kg^−1^, available phosphorus—189–280 mg·kg^−1^, available sulfur—1.2–2.6 mg·kg^−1^, available magnesium—436–790 mg·kg^−1^.

Lithuania has sufficient precipitation in all seasons, especially in the warmer months, but recently, droughts have become more frequent in the summer. The average annual precipitation in Lithuania is 600–900 mm, and evaporation is around 500 mm. The annual temperature is around 6.5–7.5° C. During the experiment, meteorological conditions varied considerably each year. Temperature data show that in the spring months (April and May) in all the years analyzed the temperature was slightly lower than the long-term average (Supplementary Fig. 2). At the beginning of summer, temperatures approached the long-term norm: in June 2020 and 2021 they were close to or slightly higher than the average, while in 2022 they were somewhat lower. The largest positive deviation was observed in July 2021 (22.6 °C), when the temperature was significantly higher than the long-term average. In August 2022 the temperature also exceeded the long-term average, whereas in 2021 it was slightly lower. Precipitation amounts, compared with the long-term conditions, showed greater variability. In April 2020, the precipitation amount was significantly lower than the long-term average. In May and August 2021, precipitation significantly exceeded the long-term average, while in June 2021 it was lower than usual. In July 2022, precipitation was higher than the long-term average, whereas in August 2022 it was considerably lower. In summary, temperature fluctuations were generally low and close to the long-term average, whereas precipitation varied much more between different years and months.

### Experimental design and agronomic operations

The stationary continuously 3-year replicated field experiment consisted of 7 treatments, 21 experimental plots in total, arranged with three replications in a randomized complete block design (RCBD). The size of the plot was 8 m^2^. Table 1 shows the list of performed treatments and their abbreviations. Maize (*Zea mays* L.) (cultivar “Pioneer” hybrid P7034), industrial hemp (*Cannabis sativa* L.) (cultivar “Austa SK”), and faba bean (*Vicia faba* L.) (cultivar “Vertigo”) as single, binary, and ternary crops, respectively, were grown to obtain the highest possible biomass yield at low-input pesticide-free conditions. Experimental plots were sown according to specially created seed distributing schemes^38^ (Supplementary Fig. 3). Sowing rates depend on the scheme and are presented in more detail in Supplementary Table 1.

**Table 1:** Experimental treatments and their abbreviations.

Biodiversity level	Crops	Abbreviation and number of treatment
Single crop	Maize, hemp, faba bean (separate crops)	M (1), H (2), FB (3)
Binary crop	Maize + hemp	M + H (4)
	Maize + faba bean	M + FB (5)
	Hemp + faba bean	H + FB (6)
Ternary crop	Maize + hemp + faba bean	M + H + FB (7)

Before setting up the experiment, oats (*Avena sativa* L.) were grown in the experimental site as pre-crop. The soil of the experiment was plowed with a Gamega PP-3-43 plow (Lithuanian producer) with semi-helical shellboards in each fall (usually in October) and cultivated with a complex Laumetris KLG-3.6 cultivator (Lithuanian producer) in spring (usually in April) before sowing. Before sowing, plots were fertilized by the mineral complex fertilizer (NPK 15:15:15. 300 kg·ha^−1^) at the total rate of nutrients N_45_P_45_K_45_. This fertilization rate is insufficient for achieving high biomass yields; however, we aim to demonstrate a more pronounced effect of continued multicropping on soil fertility. We expected that the ecological services of faba beans would compensate for the negative impact of crops continuing to grow on the soil properties. Experimental plots were sown by hand equipment The presented plant sowing schemes ensured the development of crop mixtures with minimal competition (Supplementary Fig. 4). The crops’ inter-rows were loosened 1–2 times until the crops covered the inter-rows. Crop biomass production was harvested after a short 103–105-day vegetative season, when the faba beans reached full maturity (Supplementary Table 2). At that time, the biomass of maize and hemp had not yet reached its maximum. Still, faba beans produce abundant and valuable biomass (especially in the first year of growing), which we did not want to lose because in August, there is usually a shortage of biomass for fuel production in Lithuania. The applied technological operations and technical means, and their energy and CO_2eq._ balance are discussed in more detail in our previous article^39^.

### Methods and analysis

The BBCH scale was used to assess crop development. Measurements were done during the vegetative period when faba bean began to bloom (maize BBCH 51–53, industrial hemp BBCH 60–62, faba bean BBCH 63–65), assessing plant height, leaf chlorophyll index, assimilation area, and fresh (fresh) biomass of each crop species. At the end of the plant vegetation period (maize BBCH 79–80, industrial hemp BBCH 81–83, faba bean BBCH 95–97), the fresh biomass of maize, hemp, and faba bean assessed separately, but also the total fresh and dried biomass of the crops per unit area.

For development indicator studies, five plants of each crop species were cut out in each experimental plot. The assimilation area of plant leaves (cm^2^) was determined with a leaf area meter Win Dias (“Delta-T Devices” Ltd, UK). The chlorophyll index of plant leaves was measured with a chlorophyll meter CCM–200 Plus (Opti-Sciences, Inc. Hudson, New Hampshire, USA). The height of the plants was measured, and they were weighed, thus determining their fresh biomass. At the end of faba bean vegetative period (BBCH 95–97), all crops’ samples were taken at least five spots per plot, in a 0.5 m longitudinal row. An average sample was formed. The biomass of maize and hemp stilled fresh (maize BBCH 79–80, industrial hemp BBCH 81–83). We wished the mature bean biomass to be included in the total biomass yield, rather than just being used to improve soil fertility. Of course, the maize and hemp biomass had not yet reached full maturity. Vegetative period lasted about 103–105 days from the germination of at least two crops. A total of 36 research samples were formed. To determine the dried biomass, the samples were dried in a thermostat at a temperature of 105 °C.

The produced biomass can be utilized in the energy sector and other industries, particularly to produce biofuels, fertilizers, bio-additives, and similar products. Therefore, its elemental composition is important (see Supplementary Table 3). Supplementary Table 4 also presents the main characteristics of the biofuel pellets. The chemical composition was analyzed at the laboratories of the Lithuanian Research Centre for Agriculture and Forestry in Kaunas, Lithuania.

The experimental data were analyzed using two-way ANOVA, and the treatment effect was estimated by the F-test and the least significant difference (LSD). SYSTAT software were employed. Significant differences between the treatments are marked with different lowercase letters, when *P* ≤ *0.05* > *0.01*. Significant differences between the vegetation conditions of individual years of the experimentation are marked with different uppercase letters, when *P* ≤ *0.05* > *0.01*. A correlation analysis was performed with SigmaStat software (Systat Software Inc. San Jose, California, USA).

Principal Component Analysis (PCA) was used to establish the correlation between different indicators in single, binary, and ternary crops. This analysis creates new artificial variables (principal components) based on the analysed variables. Its central premise was the ability to visualise the relationships between individual variables in a two-dimensional diagram showing the coordinate system of the first two principal components. Based on the position of the vectors in space, it is possible to determine which variables are correlated with each other. A lower angle between the vectors shows the stronger positive correlation. When the vectors are aligned on the same line but in opposite directions, there is a strong negative correlation between the variables. However, when the vectors are aligned at an angle close to 90 degrees, there is no correlation^40^.

The results of the investigations were also grouped using cluster analysis. The clustering of all tested single, binary, and ternary crops, considering the estimated average height of crops, leaf assimilation area, and chlorophyll index, crop fresh and dried biomass, was carried out according to the Ward criterion using Euclidean distance matrices. Statistical analysis was performed using the Statistica software package Statistica 10 (TIBCO Software Inc., Palo Alto, CA, USA). Calculations were performed at Vytautas Magnus University Agriculture Academy.

## Results and discussion

This chapter analyzes the growth, physiological traits, and biomass production of maize, industrial hemp, and faba bean under different cropping systems in short-growth boreal vegetative conditions. Key parameters include plant height, leaf assimilation area, chlorophyll index, fresh and total biomass. Technological, energy, and environmental aspects are also evaluated, including fuel use, net energy yield, and freshhouse gas emissions. The findings provide an integrated assessment of crop performance, interspecific interactions, and the potential for biomass utilization.

### Average plant height

The intensity of crop diversification (factor A) negatively affected the average heights of maize and industrial hemp. Maize and industrial hemp were tallest in single crops (180.8 cm and 186.7 cm) and shortest in the ternary crop (158.0 cm and 148.0 cm), while intercropping with faba bean partially increased their height (maize 175.6 cm, hemp 185.0 cm). Similarly, intercropping maize with cowpea or soybean resulted in maize heights of 177–179 cm compared to 181.8 cm in single maize, suggesting intercrop interactions can modestly influence plant height depending on species combinations and agronomic conditions^41^. Similarly, Wondire et al.^42^showed that maize plant height was significantly higher in a 2:1 maize–lablab intercropping pattern compared with single maize. The increase in height was linked to better canopy development and nutrient availability from lablab. Fisher et al.^43^ in Germany and Hirpa^44^ in Ethiopia found no effect on maize height when grown in a binary crop with faba bean. Li et al.^45^ found the negative effect. The height indicators of faba bean differed from those of maize and industrial hemp. The lowest faba bean height was observed in a single crop, with a significant difference of 10.9 cm when grown together with maize.

In contrast, faba bean grew tallest in the binary M + FB crop (101.0 cm) and shortest in single crops (90.1 cm). Differences are significant (Table 2).

**Table 2:** Effects of crop diversification level and vegetative conditions of the experimental year on the crop height of maize, industrial hemp, and faba bean (cm).

Crop diversification (factor A)	Conditions of the vegetative season (factor B)			Average A
	2020	2021	2022	
**Maize**				
**M**	246.8	183.3	112.5	**180.8a**
**M** + H	205.7	170.3	104.1	**160.0b**
**M** + FB	210.7	191.9	124.1	**175.6a**
**M** + H + FB	194.7	170.7	108.6	**158.0b**
***Average B***	**214.5A**	**179.1B**	**112.3C**	
***Interaction A x B***, *F-akt. 2.8, P* ≤ *0.05* > *0.01, R*_*05*_*–21.75, R*_*01*_*–29.56*				
**Industrial hemp**				
**H**	233.1	193.6	133.5	**186.7a**
M + **H**	204.5	156.6	111.4	**157.5bc**
**H** + FB	217.5	184.0	153.5	**185.0ab**
M + **H** + FB	175.2	145.9	122.9	**148.0c**
***Average B***	**207.6A**	**170.0B**	**130.4C**	
***Interaction A x B***, *F-akt. 0.51, P* > *0.05, R*_*05*_*–48.96, R*_*01*_*–66.55*				
**Faba bean**				
**FB**	95.9	75.1	99.3	**90.1b**
M + **FB**	111.2	89.1	102.8	**101.0a**
H + **FB**	103.9	76.5	111.1	**97.1ab**
M + H + **FB**	104.7	70.3	105.8	**93.6ab**
***Average B***	**103.9A**	**77.7B**	**104.7A**	
***Interaction A x B***, *F-akt. 0.86, P* > *0.05, R*_*05*_*–17.7, R*_*01*_*–24.06*				

M, maize single crop; H, hemp single crop; FB, faba bean single crop; M + H, binary maize and hemp crop; M + FB, binary maize and faba bean crop; H + FB, binary hemp and faba bean crop; M + H + FB, ternary maize, hemp and faba bean crop. Different lower case letters indicate significant differences between crop diversification level at *P* ≤ 0.05 > 0.01. Different upper case letters indicate significant differences between experimental year conditions at *P* ≤ 0.05 > 0.01. Significant values are in bold.

Regarding the effect of crop continuing growing (factor B) on plant height, it was found that the lowest height of maize and industrial hemp was in the last year of the experiment, i.e., the heights were significantly 1.6–1.9 times less because of degradation of most soil properties^46^.

A comprehensive assessment of the different indicators studied showed that plant height in a hemp crop was the highest expressed at crop development and productivity level compared to other experimental treatments^47^ (Supplementary Fig. 5).

Overall, crop diversification reduced the average heights of maize and industrial hemp, with the tallest plants in single crops and the shortest in ternary systems. Intercropping with faba bean partially mitigated this effect, increasing plant height, while faba bean itself grew tallest when combined with maize. A three-year continued cropping decreased maize and hemp heights, highlighting the combined influence of crop diversity and soil conditions on plant growth.

### Leaf assimilation area

The increase in the number of plant species in the crop influenced the decrease in the assimilation area of their leaves. The significantly largest leaf area of maize and industrial hemp was determined when they grew as single crops (Table 3).

**Table 3:** Effects of crop diversification level and vegetation conditions of the experimental year on the leaf assimilation area of maize, industrial hemp, and faba bean (cm^2^).

Crop diversification (factor A)	Conditions of the vegetative season (factor B)			Average A
	2020	2021	2022	
**Maize**				
**M**	362.7	427.1	235.7	**341.8a**
**M** + H	180.2	361.3	163.7	**235.1b**
**M** + FB	0231.0	445.9	156.2	**277.7ab**
**M** + H + FB	156.3	411.2	133.1	**233.5b**
***Average B***	**232.5B**	**411.4A**	**172.2B**	
***Interaction A x B***, *F-akt. 0.53, P* > *0.05, R*_*05*_*–181.09, R*_*01*_–*246.13*				
**Industrial hemp**				
**H**	95.7	218.4	135.2	**149.8a**
M + **H**	59.4	101.6	81.2	**80.7b**
**H** + FB	64.6	187.1	113.0	**121.5ab**
M + **H** + FB	73.2	219.8	108.2	**133.7a**
***Average B***	**73.2B**	**181.7A**	**109.4B**	
***Interaction A x B***, *F-akt. 0.9, P* > *0.05, R*_*05*_*–76.43, R*_*01*_*–103.88*				
**Faba bean**				
**FB**	141.9	213.6	288.1	**214.5a**
M + **FB**	83.8	225.9	371.8	**227.2a**
H + **FB**	67.7	334.4	229.6	**210.6a**
M + H + **FB**	78.6	148.4	239.7	**155.5a**
***Average B***	**93.0B**	**230.6A**	**282.3A**	
***Interaction A x B***, *F-akt. 2.0, P* > *0.05, R*_*05–*_*133.05, R*_*01*_*–180.84*				

M, maize single crop; H, hemp single crop; FB, faba bean single crop; M + H, binary maize and hemp crop; M + FB, binary maize and faba bean crop; H + FB, binary hemp and faba bean crop; M + H + FB, ternary maize, hemp and faba bean crop. Different lower case letters indicate significant differences between crop diversification level at *P* ≤ 0.05 > 0.01. Different upper case letters indicate significant differences between experimental year conditions at *P* ≤ 0.05 > 0.01. Significant values are in bold.

When maize was grown in a binary crop with faba bean, its leaf assimilation area was 16% higher compared to other diversified crops. This likely occurred because faba bean competes less with maize for resources and enriches the soil with nitrogen, an essential nutrient that supports maize growth. Similarly, Li et al.^48^ and Yang et al.^49^ also found that the maize-soybean binary crop increased the assimilation area of maize leaves. Yang et al.^50^ and Corre-Hellou et al.^51^ confirmed these results with pea (*Pisum sativum* L.) cover- and inter-crops. A comprehensive data analysis revealed that maize leaves’ assimilation area was the highest at crop development and productivity levels compared to other experimental treatments (Supplementary Fig. 5). Additionally, we found a positive moderate correlation between maize leaf assimilation area and plant height (r = 0.64, *P* ≤ 0.050 > 0.010).

The largest leaf assimilation area of hemp was found in the binary crop with faba bean, and the highest area of faba bean leaves was found in the binary crop with maize. On the contrary, Wu et al.^52^ found that when faba bean grows together with maize, increased competition for sunlight causes faba bean stems to lengthen and reduces their leaf assimilation area.

Vegetative season conditions (factor B) strongly influenced leaf assimilation area. Maize and industrial hemp had the highest leaf areas in 2021 (411.4 cm^2^ and 181.7 cm^2^, respectively) and the lowest in 2022 (172.2 cm^2^ and 109.4 cm^2^), while faba bean reached its largest leaf area in 2022 (282.3 cm^2^) compared to 2020 (93.0 cm^2^). These differences were significant across years, indicating that favorable vegetative conditions in 2021–2022 enhanced leaf growth, while the effects were consistent across crop diversification levels (A × B interactions not significant).

Generally, increasing crop diversity generally reduced the leaf assimilation area of maize and industrial hemp, which was largest in single crops. Intercropping with faba bean increased leaf areas for maize and hemp, while faba bean leaves were largest when grown with maize. Vegetative conditions also influenced leaf assimilation, with yearly variations affecting all three species.

### Chlorophyll index of plant leaves

The chlorophyll index of leaves of each single crop was usually higher than that of mixed crops, but mainly not significant in hemp crop (Table 4). Peñafiel–Sandova^53^ also found the highest chlorophyll index in the maize single crop without concurrence from companions. Opposite results found^54^. In pea-intercropped winter wheat, the leaf area increased by about 10–20%, and the chlorophyll index increased by approximately 5–15% compared with wheat grown as a single crop. Similarly^55^, highlighted that maize grown together with legumes such as soybean or faba bean showed 5–20% higher chlorophyll content (SPAD values) and 10–25% higher leaf area index (LAI) compared with maize grown as a sole crop. Improved canopy structure in these intercrops also increased light interception by about 10–15%, which enhanced photosynthetic activity and biomass accumulation.

**Table 4:** Effects of crop diversification level and vegetative conditions of the experimental year on the leaf chlorophyll index of maize, industrial hemp, and faba bean.

Crop diversification (factor A)	Conditions of the vegetative season (factor B)			Average A
	2020	2021	2022	
**Maize**				
**M**	39.7	27.8	7.4	**25.0a**
** M** + H	24.2	23.8	7.6	**18.6b**
**M** + FB	28.8	36.4	9.9	**25.0a**
**M** + H + FB	25.9	27.1	9.9	**20.9ab**
***Average B***	**29.7A**	**28.8A**	**8.7B**	
***Interaction A x B***, *F-akt. 4.29, P* ≤ *0.010* > *0.001, R*_*05*_*–6.99, R*_*01*_*–9.5*				
**Industrial hemp**				
**H**	23.1	30.2	16.7	**23.4a**
M + **H**	20.4	27.2	17.4	**21.7a**
**H** + FB	22.4	32.9	17.2	**24.2a**
M + **H** + FB	24.2	24.5	14.8	**21.2a**
***Average B***	**22.5B**	**28.7A**	**16.5C**	
***Interaction A x B***, *F-akt. 0.9, P* > *0.05, R*_*05*_*–7.3, R*_*01*_*–9.92*				
**Faba bean**				
**FB**	27.5	25.2	54.4	**35.7a**
M + **FB**	22.7	20.9	39.8	**27.8b**
H + **FB**	20.2	21.0	41.0	**27.4b**
M + H + **FB**	25.6	22.8	47.9	**32.1ab**
***Average B***	**24.0B**	**22.5B**	**45.8A**	
***Interaction A x B***, *F-akt. 0.86, P* > *0.05, R*_*05*_*–8.31, R*_*01*_*–11.3*				

M, maize single crop; H, hemp single crop; FB, faba bean single crop; M + H, binary maize and hemp crop; M + FB, binary maize and faba bean crop; H + FB, binary hemp and faba bean crop; M + H + FB, ternary maize, hemp and faba bean crop. Different lower case letters indicate significant differences between crop diversification level at *P* ≤ 0.05 > 0.01. Different upper case letters indicate significant differences between experimental year conditions at *P* ≤ 0.05 > 0.01. Significant values are in bold.

In our experiment, according to the average data of our experiment, factor B, the chlorophyll index in the last year of the experiment decreased significantly 3 times in maize and 1.4 times in industrial hemp crops compared to the first year of study. The opposite results were obtained in faba bean crops, since the chlorophyll index was significantly highest in the third year of the study.

According to the comprehensive analysis of the research data, the crop leaf chlorophyll index was the most common in the ternary crop. In another tested crops, the rating of this index mainly did not exceed the evaluation threshold (5.0) (Supplementary Fig. 5).

### Fresh biomass of individual plant species

An interaction was found between the effect of crop diversification and vegetative conditions of individual research years on the average fresh biomass of plants at the end of the vegetation. Crop diversification (factor A) negatively affected the fresh biomass of individual plants, since it was highest in single crops of maize, industrial hemp, and faba bean (Tables 5 and 6). At the middle of vegetative season, differences were significant in hemp crop only. Among the diversified crops, the highest fresh biomass of maize and industrial hemp was found when they grew together with faba bean. Here, the fresh biomass of maize was 3 times higher, and that of industrial hemp was 2 times higher than in the ternary crop. Combining maize with various legumes, such as faba bean, improved maize crop growth, increased its fresh biomass, and increased plant quality^56^. The fresh biomass of faba bean did not differ significantly in the diversified crops. The vegetative conditions (factor B) of the research years also had a significant impact on the fresh biomass of tested crops. The highest fresh biomass of these plants was in the first year of the experiment, and then it consistently decreased. In the third year of the experiment, the fresh biomass of maize, hemp, and faba bean significantly decreased by 2–4 times.

**Table 5:** Effects of crop diversification level and vegetative conditions of the experimental year on the average fresh biomass of maize, industrial hemp, and faba bean in the middle of vegetative season (g m^−2^).

Crop diversification (factor A)	Conditions of the vegetative season (factor B)			Average A
	2020	2021	2022	
**Maize**				
**M**	133.6	153.6	115.8	**134.3a**
**M** + H	70.7	55.7	82.9	**69.8c**
**M** + FB	70.9	159.4	77.1	**102.4b**
**M** + H + FB	43.4	93.9	57.9	**65.0c**
***Average B***	**79.6B**	**115.6A**	**83.4B**	
***Interaction A x B***, *F-akt. 2.31, P* > *0.05, R*_*05*_*–50.99, R*_*01*_*–69.31*				
**Industrial hemp**				
**H**	26.7	104.9	58.3	**63.3a**
M + **H**	20.5	94.9	35.4	**50.3a**
**H** + FB	22.7	89.8	58.3	**57.0a**
M + **H** + FB	15.9	96.0	48.0	**53.3a**
***Average B***	**21.4C**	**96.4A**	**50.0B**	
***Interaction A x B***, *F-akt. 0.67, P* > *0.05, R*_*05*_*–23.19, R*_*01*_*–31.52*				
**Faba bean**				
**FB**	59.0	135.6	146.7	**113.7a**
M + **FB**	45.8	130.5	143.8	**106.7a**
H + **FB**	31.8	131.1	121.7	**94.8a**
M + H + **FB**	39.6	113.6	147.9	**100.4a**
***Average B***	**44.0B**	**127.7A**	**140.0A**	
***Interaction A x B***, *F-akt. 0.62, P* > *0.05, R*_*05*_*–35.1, R*_*01*_*–47.71*				

M, maize single crop; H, hemp single crop; FB, faba bean single crop; M + H, binary maize and hemp crop; M + FB, binary maize and faba bean crop; H + FB, binary hemp and faba bean crop; M + H + FB, ternary maize, hemp and faba bean crop. Different lower case letters indicate significant differences between crop diversification level at *P* ≤ 0.05 > 0.01. Different upper case letters indicate significant differences between experimental year conditions at *P* ≤ 0.05 > 0.01. Significant values are in bold.

**Table 6:** Effects of crop diversification level and vegetation conditions of the experimental year on the average fresh biomass of maize, industrial hemp, and faba bean at the end of vegetative season (g m^−2^).

Crop diversification(factor A)	Conditions of the vegetative season (factor B)			Average A
	2020	2021	2022	
**Maize**				
**M**	7335.6	4163.3	1733.3	**4410.7a**
**M** + H	2450.0	1810.0	920.8	**1726.9c**
**M** + FB	4137.8	3483.3	1291.1	**2970.7b**
**M** + H + FB	1060.0	1540.6	357.8	**986.1d**
***Average B***	**3745.8A**	**2749.3B**	**1075.8C**	
***Interaction A x B***, *F-akt. 11.05, P* ≤ *0.010* > *0.001, R*_*05*_*–989.94, R*_*01*_*–1345.51*				
**Industrial hemp**				
**H**	3022.2	1791.1	775.6	**1863.0a**
M + **H**	1323.3	644.2	260.0	**742.5b**
**H** + FB	2204.4	1787.2	596.1	**1529.3a**
M + **H** + FB	1146.7	619.4	281.7	**682.6b**
***Average B***	**1924.2A**	**1210.5B**	**478.3C**	
***Interaction A x B****, F-akt. 1.16, P* > *0.05, R*_*05*_*–932.49, R*_*01*_*–1267.41*				
**Faba bean**				
**FB**	1128.9	822.2	448.9	**800.0a**
M + **FB**	693.1	333.3	336.7	**454.4b**
H + **FB**	420.0	315.6	323.3	**353.0b**
M + H + **FB**	684.4	288.9	365.6	**446.3b**
***Average B***	**731.6A**	**440.0B**	**368.6B**	
***Interaction A x B***, *F-akt. 5.12, P* ≤ *0.010* > *0.001, R*_*05*_*–18.45, R*_*01*_*–245.27*				

M, maize single crop; H, hemp single crop; FB, faba bean single crop; M + H, binary maize and hemp crop; M + FB, binary maize and faba bean crop; H + FB, binary hemp and faba bean crop; M + H + FB, ternary maize, hemp and faba bean crop. Different lower case letters indicate significant differences between crop diversification level at *P* ≤ 0.05 > 0.01. Different upper case letters indicate significant differences between experimental year conditions at *P* ≤ 0.05 > 0.01. Significant values are in bold.

Maize fresh biomass was partly dependent on its leaf chlorophyll index (r = 0.58, *P* ≤ 0.050 > 0.010) and leaf assimilation area (r = 0.72, *P* ≤ 0.050 > 0.010). Industrial hemp dependencies were similar (r = 0.87, r = 0.86, *P* ≤ 0.010 > 0.001). A statistically significant correlation was also established between faba bean leaf assimilation area and capacity of fresh biomass (r = 0.87, *P* ≤ 0.010 > 0.001). Plants’ fresh biomass also partly depended on the crop density and average height of plants.

Fresh biomass of maize, industrial hemp, and faba bean was highest in single crops and generally decreased with increased crop diversification. Among mixed crops, maize and hemp produced the most biomass when grown with faba beans, while faba bean biomass remained largely unaffected. Yearly vegetative conditions and plant traits such as leaf area, chlorophyll index, and height also significantly influenced biomass accumulation.

### Total biomass of cultivations

This chapter focuses on the total crop biomass production of different cropping systems and how it is influenced by crop diversification and vegetative conditions over three years. It describes how single, binary, and ternary crop combinations affect the accumulation of fresh and dried biomass, with intercropping—especially maize and hemp with faba bean—leading to higher biomass than single crops. The chapter also analyzes relationships between plant traits and biomass using Principal Component Analysis (PCA), showing how height, leaf area, and chlorophyll index influence biomass differently across years, and cluster analysis, which highlights which crop combinations are most effective for biomass production. It integrates these analyses to explain how crop type, physiological traits, and environmental conditions collectively determine biomass yield.

#### Effects of crop diversification and vegetative conditions on the total dried biomass of cultivation

An interaction between factors (crop diversification and the vegetative conditions) of individual experimental years on the total fresh biomass of crops at the end of the plant vegetative season was established. Among single crops, the significantly highest fresh biomass per area was observed for maize (4410.7 g m^−2^). Among diversified crops, the highest significant fresh biomass was obtained when growing a binary crop of maize and faba bean (3421.5 g m^−2^). Shtaya et al.^57^ also confirmed that faba bean, when mixed with other companions, increased the total fresh biomass of the crop. Streit et al.^58^ found that faba bean in mixtures produces an average of 5% more fresh biomass than in single crops only. In our experiment, the fresh biomass of the ternary crop was 25% lower than that of the binary crop of maize and faba bean (Table 7).

**Table 7:** Effects of crop diversification level and vegetative conditions of the experimental year on the average fresh biomass of crops (g m^−2^).

Crop diversification(factor A)	Conditions of the vegetative season (factor B)			Average A
	2020	2021	2022	
M	7335.6	4148.9	1760.0	**4410.7a**
H	3022.2	1791.1	775.6	**1863.0d**
FB	1128.9	822.2	448.9	**800.0e**
M + H	3773.3	2454.2	1180.8	**2469.4c**
M + FB	4820.0	3816.7	1627.8	**3421.5b**
H + FB	2620.0	2086.1	917.2	**1874.4d**
M + H + FB	3563.3	2737.8	1370.6	**2557.2c**
***Average B***	**3751.9A**	**2551.0B**	**1154.4C**	
***Interaction A x B***, *F-akt. 4.85, P* ≤ *0.010* > *0.001, R*_*05*_*–1019.97, R*_*01*_*–1364.86*				

M, maize single crop; H, hemp single crop; FB, faba bean single crop; M + H, binary maize and hemp crop; M + FB, binary maize and faba bean crop; H + FB, binary hemp and faba bean crop; M + H + FB, ternary maize; hemp and faba bean crop. Different lower case letters indicate significant differences between crop diversification level at *P* ≤ 0.05 > 0.01. Different upper case letters indicate significant differences between experimental year conditions at *P* ≤ 0.05 > 0.01. Significant values are in bold.

The vegetative conditions (factor B) of individual experimental years also had a significant effect on the fresh biomass of crops at the end of the vegetative period. It was significantly highest in the first year of the experiment and decreased by 30% in the second and third experimental years.

As expected, the ternary crop had the highest total dried biomass, which was significantly 8 times higher than that of the single maize crop (Table 8), because of the time of biomass harvesting, maize contained a large amount of water, while the faba bean biomass was close to air-dried (BBCH 95–97) and had highest productivity level. According to Ciampitti et al.^59^, faba bean harvesting at the right time is very important not only to ensure the best crop yield but also to maximize biomass energy yield. Faba bean-mixed crops can also increase not total yield only but reduce crop weed and disease infestation, increase land use efficiency, and sustainability^57,60^. Moreover, in our experiment, the dried biomass of all single crops was lower compared to the biomass of diversified crops (Table 8). Dzvene^61^ obtained similar results. Bybee–Finley et al.^62^ found that in an experiment with millet, sorghum, and hemp, in the first year of crop cultivation, the biomass of plants grown in mixtures was higher than that of single crops.

**Table 8:** Effects of crop diversification level and vegetative conditions of the experimental year on the average dried biomass of crops (g m^−2^).

Crop diversification (factor A)	Conditions of the vegetative season (factor B)			Average A
	2020	2021	2022	
M	446.1	184.8	88.7	**239.9d**
H	903.9	133.2	168.9	**402.0d**
FB	981.1	235.7	245.0	**487.3** **cd**
M + H	2470.2	240.4	158.2	**956.2b**
M + FB	1716.6	247.5	279.2	**747.8bc**
H + FB	2005.7	231.0	263.8	**833.5b**
M + H + FB	4401.6	412.9	789.7	**1868.0a**
***Average B***	**1846.4A**	**240.8B**	**284.8B**	
***Interaction A x B***, *F-akt. 11.38, P* ≤ *0.010* > *0.001, R*_*05*_*–584.52, R*_*01*_*–782.16*				

M, maize single crop; H, hemp single crop; FB, faba bean single crop; M + H, binary maize and hemp crop; M + FB, binary maize and faba bean crop; H + FB, binary hemp and faba bean crop; M + H + FB, ternary maize, hemp and faba bean crop. Different lower case letters indicate significant differences between crop diversification level at *P* ≤ 0.05 > 0.01. Different upper case letters indicate significant differences between experimental year conditions at *P* ≤ 0.05 > 0.01. Significant values are in bold.

Overall, in our experiment, the crops’ 3-year continued growing decreased the total biomass yields. Only in the first year of the experiment did we receive the highest yields (especially in the ternary crop), and in the second and last years of the study, they decreased by about 6.5 times due to the degradation of soil properties, especially depletion of nitrogen content. Across the multi‑cropping treatments, total nitrogen decreased in most cases, especially in systems with high biomass production such as the ternary maize–hemp–faba bean mixture^63^.

#### Statistical analysis

After conducting a comprehensive assessment of the experimental data, it was found that the ternary crop had the highest comprehensive evaluation index (CEI) value (4.54); therefore, it was the most effective in growing plant total biomass. The formation of higher dried biomass in the ternary crop was most influenced by the higher leaf chlorophyll index (Supplementary Fig. 5, marked in yellow). A higher CEI value (4.12) was also obtained in binary M + H cultivation. This confirms the statement of Branca et al.^64^ and Hu et al.^65^ that maize and industrial hemp are the most promising crops in the field of biomass processing.

**Fig. 1:**
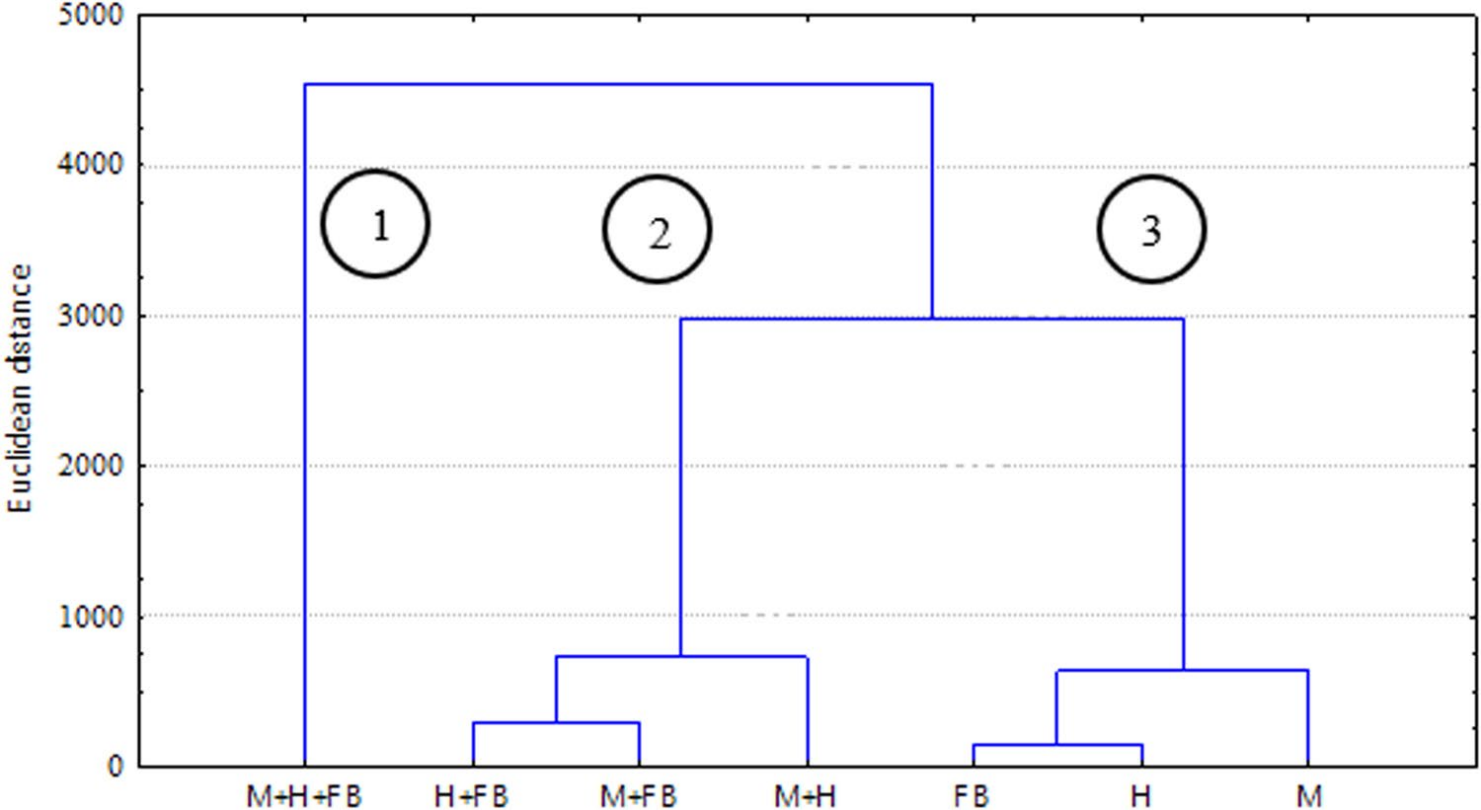
Hierarchical cluster analysis (Ward method, Euclidean distance matrices) of single, binary, and ternary crops considering the dried biomass. Note: PH, plant height; LA,leaf area; ChI, chlorophyll index in the leaves; GB1, fresh biomass in the middle of vegetative season; GB2, fresh biomass at the end of vegetative season.

**Fig. 2:**
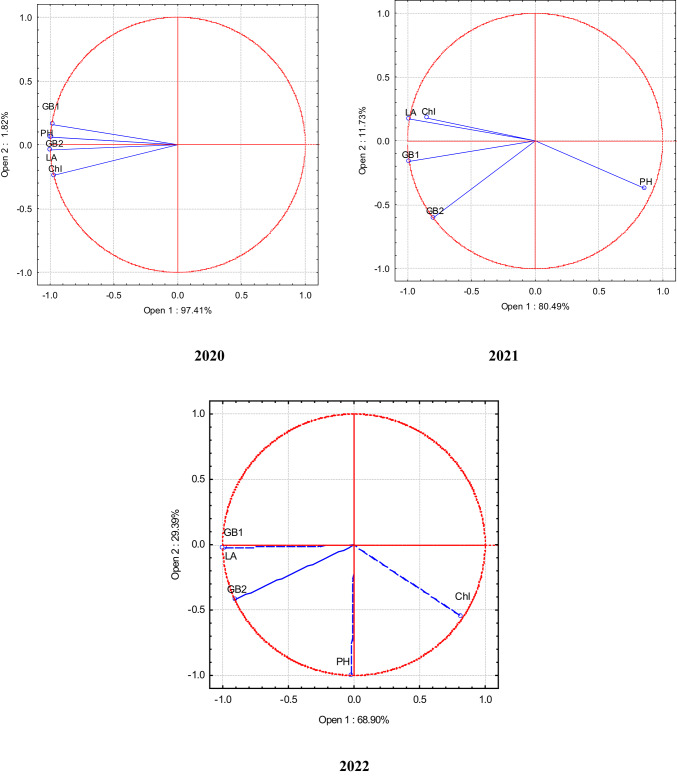
PCA of maize in the single, binary, and ternary crops. 2020–2022.Note: PH, plant height; LA,leaf area; ChI, chlorophyll index in the leaves; GB1, fresh biomass in the middle of vegetative season; GB2, fresh biomass at the end of vegetative season.

A generalized cluster analysis of our experimental data showed that maize and hemp were more efficiently grown in intercropping with faba beans due to their higher biomass capacity (Fig. 1). The PCA was also done for different species of companion crops in cultivation:

*Maize* In 2020, the aboveground biomass of maize in the middle and end of the vegetative season was closely related to plant height and plant photosynthetic indicators ˗ plant leaf assimilation area and chlorophyll index in leaves (Fig. 2). In 2021, the aboveground biomass of maize in the middle of the vegetation correlated with plant leaf assimilation area and chlorophyll index in leaves. The latter indicators had less influence on the aboveground biomass of plants at the end of the vegetation. In 2022, similar trends were found as in 2021.

**Fig. 3:**
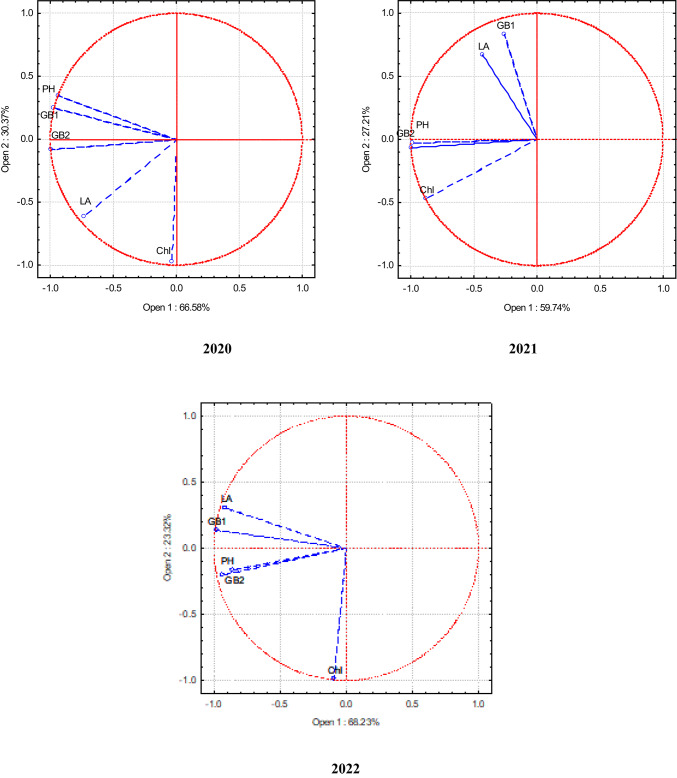
PCA of technical hemp in the single, binary, and ternary crops. 2020–2022. Note: PH, plant height; LA,leaf area; ChI, chlorophyll index in the leaves; GB1, fresh biomass in the middle of vegetative season; GB2, fresh biomass at the end of vegetative season.

*Industrial hemp* In 2020, the aboveground biomass of hemp in the middle and end of the vegetative period was most influenced by plant average height. The influence of leaf assimilation area was lower (Fig. 3). In 2021, two groups of closely correlated indicators emerged: the first group consisted of the aboveground biomass of plants at the beginning of the vegetative period and the leaf assimilation area, and the second group consisted of the aboveground biomass of plants at the end of the vegetative period and plant height. In 2022, the aboveground biomass of hemp in the middle of the vegetative season was closely related to the plant leaf assimilation area. The aboveground biomass at the end of the vegetative period was more dependent on the plant height.

**Fig. 4:**
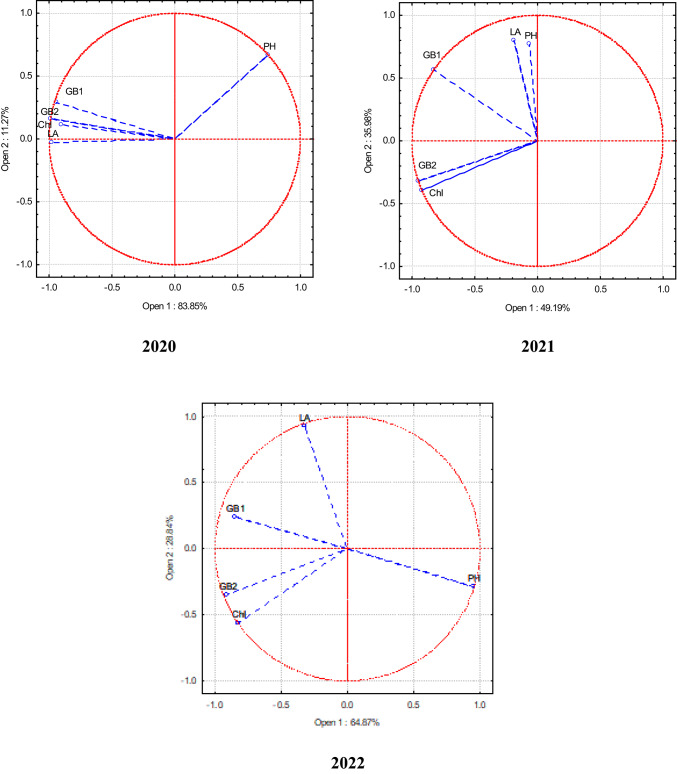
PCA of faba bean in the single, binary, and ternary crops. 2020–2022.

*Faba bean* In 2020, the aboveground biomass of faba beans in the middle and end of the vegetative period was closely related to plant photosynthetic indicators—plant leaf assimilation area and chlorophyll index (Fig. 4). In 2021, the aboveground biomass of faba beans at the middle of the vegetative period was less influenced by plant height and photosynthetic indicators. In 2022, the aboveground biomass of faba beans at the end of the vegetative period depended most on the chlorophyll concentration in the leaves.

In 2020, both middle- and end-season biomass (GB1, GB2) were strongly associated with plant height (PH), leaf area (LA), and chlorophyll index (ChI). In 2021–2022, middle-season biomass remained linked to leaf traits, while end-season biomass depended more on plant height, indicating that maize biomass is influenced by both morphological and physiological traits, with their relative effects shifting across years.

Maize produced the highest fresh biomass among single crops, while the binary combination of maize and faba bean increased biomass by about 25% compared with the ternary crop. PCA showed that maize middle-season biomass was strongly associated with leaf assimilation area and chlorophyll index, while end-season biomass depended more on plant height; hemp end-season biomass was mainly influenced by height, and faba bean biomass was closely linked to chlorophyll content. Cluster analysis highlighted that maize and hemp intercropped with faba bean had 20–30% higher biomass than other combinations, indicating the efficiency of these mixtures. Vegetative conditions significantly affected biomass, with the first experimental year yielding up to 30% more than subsequent years due to soil degradation. So, it is recommended to grow low-fertilized and pesticide-free ternary crops in existing on-farm crop rotations for one year only. Continuing crops need to be well fertilized, because biomass productivity gradually decreases. Aubin et al.^66^ also concluded that higher N rates can maximally improve hemp growth, plant height, and biomass.

### Technological, energy, and environmental aspects

Efficient crop production relies not only on selecting suitable species but also on optimizing cultivation practices, including soil preparation, sowing, fertilization, and crop management techniques. Diversified cropping systems, such as intercropping and mixed-species plantings, have the potential to enhance resource use, improve biomass yield, and contribute to sustainable agriculture. In addition to agronomic performance, evaluating energy efficiency, greenhouse gas emissions, and potential use of crop biomass for renewable energy is increasingly important for assessing the overall sustainability of different cropping strategies. These considerations highlight the need to integrate crop management, environmental impact, and biomass utilization when designing modern agricultural systems.

All tested cropping technologies include stubble cultivation (depth 12–15 cm) in fall, deep plowing before wintering, pre-sowing cultivation, and fertilization. One-pass conventional sowing was used for single crops, while two-pass ternary sowing was used for binary and ternary crops. Inter-row loosening (2–3 cm depth) was performed twice for all cultivations, except the ternary crop. In the ternary crop, the inter-row loosening was performed once due to higher crop densities. In calculations, we simulated one-pass biomass harvesting (low harvester load) for M, FB, and M + FB crops, one-pass biomass harvesting (high harvester load) for H, M + H, and H + FB crops, and two-pass biomass harvesting (high harvester load) for the ternary crop. Tractor power (kW) for calculations ranged from 45–67 kW (for sowing, fertilization, and shallow loosening) to 102 kW (for deeper tillage operations). The harvester power remained constant at 250 kW. We adjusted the field capacity from 1.82 to 0.68 ha h^−1^ harvester load. Technological aspects are discussed in more detail in Romaneckas et al.^39^.

The highest consumption of fuel was calculated for the ternary crop (M + H + FB) (103.3 L ha^−1^) due to a higher number and more powerful operations (Supplementary Table 5). Maize and faba bean single cropping used the lowest amount of fuel. Although the technology for growing the ternary crop required more energy input, the yield obtained compensated for this. So, the highest net energy (367,668.1 MJ ha^−1^) was also obtained in the ternary crop. Obviously, growing more plant companions in the same crop results in higher net energy^67^.

The lowest total CO_2eq._ (greenhouse gases) emissions were calculated for single hemp and faba bean cultivations, and the highest were for M + H and M + FB binary crops. GHG emission of ternary crop was average (1541.90 kg ha^−1^ CO2_eq._)^39^.

The short-growing biomass of the tested maize-hemp-faba bean ternary crop can be effectively used for energy purposes and crop fertilization, because the pH is close to neutral, contains about 1% of nitrogen and potassium, and about 0.2% of phosphorus (Supplementary Table 2), and the average yield of total dried biomass reaches almost 20 t ha^−1^. A National invention patent had been registered to produce pellets from the biomass of the ternary crop discussed^36^. These pellets had a higher density than those made from biomass of other cultivations, one of the lowest ash contents, and the highest ash shrinkage starting temperature. They met the requirements of the ISO 17225–6:2021 standard (Supplementary Table 3). Moreover, the burning of pellets in household low-power boilers did not have negative environmental consequences^68^. Unlike the evaluation of crop production technologies, the Life Cycle Assessment (LCA) of the impact of pellet disposition and use (transportation, heat production, and ash utilization) on abiotic depletion, global warming potential, acidification, and eutrophication indices showed that binary M + H crops had the lowest impact on the environment. Pellets made from ternary crop biomass had an average dimension^69^.

To summarize, all cropping systems required standard soil preparation, sowing, and fertilization, with inter-row loosening and biomass harvesting adjusted for crop type and density. Fuel use was highest in the ternary maize–hemp–faba bean crop, but it also produced the highest net energy, demonstrating that mixed crops can increase energy yield. Greenhouse gas emissions were lowest for single hemp and faba bean crops, with the ternary crop showing intermediate impact. The biomass of the ternary crop is suitable for energy production and fertilization, producing high-quality pellets that meet ISO standards and have minimal environmental consequences.

## Conclusions

Due to the eco-service of faba beans, mixtures with maize and industrial hemp showed 14% greater height, 24% higher leaf assimilation area, and 19% higher chlorophyll index. Increasing species diversity in the mixtures decreased biomass per species but increased total plot biomass. In the first year, a ternary maize–hemp–faba bean crop produced 4–8 times more dried biomass than single-species crops. Continuation over the next two years reduced yields by up to 11 times. Ternary cropping achieved the highest CEI (4.54), mainly influenced by leaf chlorophyll. It was the most fuel-intensive system (18–32% higher, 103.3 L ha^**−1**^) but also had the highest net energy (367,668.1 MJ ha^−1^) due to abundant biomass. Pellets from ternary crops met standards, with the highest density (1238 kg m^**−3**^), low ash (6%), and highest ash shrinkage temperature (1042 °C). LCA showed environmental impacts varied by cropping system; single maize pellets had the highest global warming potential (29.1 kg CO_2_ eq GJ^−1^), whereas mixed crop biomass, especially maize–hemp and hemp–faba bean, had lower impacts in global warming, acidification, eutrophication, and resource depletion. These results demonstrate that diversified cropping systems can increase bioenergy yield and reduce environmental burdens, improving sustainability.

## Supplementary Information

Below is the link to the electronic supplementary material.

Supplementary Material 1

## Data Availability

Described data and materials are available from the corresponding author upon request. The methodology for the comprehensive assessment and a detailed calculation example is provided in our previous article Kimbirauskienė R, Sinkevičienė A, Švereikaitė A, Romaneckas K. The Complex Effect of Different Tillage Systems on the Faba Bean Agroecosystem. Plants. 2024; 13(4):513. 10.3390/plants13040513, Supplementary materials, Table S1. The meteorological indicators presented in the article were obtained from the Lithuanian Hydrometeorological Service, Kaunas Meteorological Station. [https://www.meteo.lt/en/organization/structure-and-contacts/contacts/] in Supplementary materials, Figure 2. Data on main characteristics of grown multi crop solid fuel pellets was referred from Petlickaitė, R., Jasinskas, A., Domeika, R., Pedišius, N., Lemanas, E., Praspaliauskas, M., Kukharets, S. Evaluation of the Processing of Multi-Crop Plants into Pelletized Biofuel and Its Use for Energy Conversion, Processes. 11(2), 421, 10.3390/pr11020421 (2023) in Supplementary materials, Table 3.

## References

[1] Oishy, M. N. et al. Unravelling the effects of climate change on the soil-plant-atmosphere interactions: A critical review. *SEH*10.1016/j.seh.2025.100130 (2025).

[2] Erdiwansyah, A. G. et al. Prospects for renewable energy sources from biomass waste in Indonesia. *Case Stud. Chem. Environ. Eng.* **10**, 100880. 10.1016/j.cscee.2024.100880 (2024).

[3] Roy, R. et al. Steam explosion treated biomass as a renewable fuel source: A review from collection to combustion. *Fuel* **378**, 132883. 10.1016/j.fuel.2024.132883 (2024).

[4] Licata, M. et al. A comparative study to assess the production of two oilseed crops (Brassica carinata A. Braun and Carthamus tinctorius L.) and the energy potential of their agricultural biomass residues. *Heliyon* **10**(22), e38654. 10.1016/j.heliyon.2024.e38654 (2024). 39584105 10.1016/j.heliyon.2024.e38654 PMC11583710

[5] Jasinskas, A., Petlickaitė, R., Praspaliauskas, M., Romaneckas, K., & Sinkevičienė, A. Impact of ash obtained after multi-crop pellet burning on spring barley fertilization. In *Engineering for Rural Development: 23rd International Scientific Conference, ERDev 2024-Proceedings*. Latvia University of Life Sciences and Technologies, **23**, pp. 644–649 (2024).

[6] Muthulakshmi, C., Sivaranjani, R. & Selvi, S. Modification of sesame (Sesamum indicum L.) for Triacylglycerol accumulation in plant biomass for biofuel applications. *Biotechnol. Rep.* **32**, e00668. 10.1016/j.btre.2021.e00668 (2021). 10.1016/j.btre.2021.e00668 PMC8449027 34567983

[7] Wang, T. et al. From intercropping to monocropping: The effects of *Pseudomonas* strain to facilitate nutrient efficiency in peanut and soil. *Plant Physiol. Biochem.* **219**, 109378. 10.1016/j.plaphy.2024.109378 (2025). 39647229 10.1016/j.plaphy.2024.109378

[8] Kumar, R. K. S., Sasikumar, R. & Dhilipkumar, T. Exploiting agro-waste for cleaner production: A review focusing on biofuel generation, bio-composite production, and environmental considerations. *J. Clean. Prod.* **435**, 140536. 10.1016/j.jclepro.2023.140536 (2024).

[9] Alcon, F. et al. Cost benefit analysis of diversified farming systems across Europe: Incorporating non-market benefits of ecosystem services. *Sci. Total Environ.* **912**, 169272. 10.1016/j.scitotenv.2023.169272 (2024). 38141994 10.1016/j.scitotenv.2023.169272

[10] Hashakimana, L., Tessema, T., Niyitanga, F., Cyamweshi, A. R. & Mukuralinda, A. Comparative analysis of monocropping and mixed cropping systems on selected soil properties, soil organic carbon stocks, and simulated maize yields in drought-hotspot regions of Rwanda. *Heliyon* **9**, e19041. 10.1016/j.heliyon.2023.e19041 (2023). 37662738 10.1016/j.heliyon.2023.e19041 PMC10474427

[11] Léonidas, H. et al. Monocropping vs mixed cropping systems under a changing climate: Smallholder farmers’ perceptions and farm profitability in Eastern Rwanda. *Environ. Sustain. Indic.* **24**, 100527. 10.1016/j.indic.2024.100527 (2024).

[12] Su, B., Liu, X., Cui, L., Xiang, B. & Yang, W. Suppression of weeds and increases in food production in higher crop diversity planting arrangements: A case study of relay intercropping. *Crop Sci.* **58**(4), 1729–1739. 10.2135/cropsci2017.11.0670 (2018).

[13] Tariq, A. et al. Combining different species in restoration is not always the right decision: Monocultures can provide higher ecological functions than intercropping in a desert ecosystem. *J. Environ. Manag.* **357**, 120807. 10.1016/j.jenvman.2024.120807 (2024). 10.1016/j.jenvman.2024.120807 38569266

[14] Chimonyo, V. G. P., et al. Chapter 18 - Yield and water use gaps in cereal multicrop systems in sub-Saharan Africa under climate change. Food Processing Technology. Principles and Practice. A volume in Woodhead Publishing Series in Food Science, Technology and Nutrition, p. 313–329 (2021).

[15] Vanino, S. et al. A comprehensive assessment of diversified cropping systems on agro-environmental sustainability in three Mediterranean long-term field experiments. *Eur. J. Agron.* **140**, 126598. 10.1016/j.eja.2022.126598 (2022).

[16] Mamimin, C., O-Thong, S. & Reungsang, A. Enhancing biogas production from hemp biomass residue through hydrothermal pretreatment and co-digestion with cow manure: Insights into methane yield, microbial communities, and metabolic pathways. *J. Environ. Manag.* **370**, 123039. 10.1016/j.jenvman.2024.123039 (2024). 10.1016/j.jenvman.2024.123039 39461148

[17] Vávrová, K. et al. Economic evaluation of hemp’s (*Cannabis sativa*) residual biomass for production of direct energy or biochar. *Fuel* **329**, 125435. 10.1016/j.fuel.2022.125435 (2022).

[18] Salentijn, E. M. J., Zhang, Q., Amaducci, S., Yang, M. & Trindade, L. M. New developments in fiber hemp (Cannabis sativa L.) breeding. *Ind. Crops Prod.* **68**, 32–41. 10.1016/j.indcrop.2014.08.011 (2015).

[19] Eaker, J. T. Influence of industrial hemp on soil health parameters in a Kentucky cropping system. Theses and Dissertations—Plant and Soil Sciences. 10.13023/etd.2025.425 (2025).

[20] Small, E. & Marcus, D. Hemp: A new crop with new uses for North America. *Trends Plant Sci.* **7**(8), 409–415. 10.1016/S1360-1385(02)02344-0 (2002).

[21] Clarke, R. C. & Merlin, M. D. *Cannabis: Evolution and ethnobotany* (University of California Press, 2013).

[22] Food and Agriculture Organization of the United Nations. Global hemp production and use: Trends and statistics. FAO, https://www.fao.org/statistics/en (2019).

[23] Grabowska, L., Rebarz, M. & Chudy, M. Breeding and cultivation of industrial hemp in Poland. *Herba Pol.* **55**(3), 243–251 (2009).

[24] Pancaldi, F., Salentijn, E. M. J. & Trindade, L. M. From fibers to flowering to metabolites: Unlocking hemp (*Cannabis sativa*) potential with the guidance of novel discoveries and tools. *J. Exp. Bot.* **76**(1), 109–123. 10.1093/jxb/erae405 (2025). 39324630 10.1093/jxb/erae405 PMC11659183

[25] Chaowana, P. et al. Utilization of hemp stalk as a potential resource for bioenergy. *Mat. Sci. En. Tech.* **7**, 19–28. 10.1016/j.mset.2023.07.001 (2024).

[26] Tripathi, M. et al. Conversion technologies for valorization of hemp lignocellulosic biomass for potential biorefinery applications. *Sep. Purif. Technol.* **320**, 124018. 10.1016/j.seppur.2023.124018 (2023).

[27] Statistics Lithuania. Agricultural statistics 2024. Official Statistics Portal, https://osp.stat.gov.lt/services-portlet/pub-edition-file?id=43060 (2024).

[28] Kaur, G. & Kaur, P. Multi-dimensional maize biomass utilization: Mitigating existing agro-waste by meaningful value addition. *J. Environ. Manag.* **320**, 126232. 10.1016/j.jenvman.2025.126232 (2025). 10.1016/j.jenvman.2025.126232 40554881

[29] Kamusoko, R. & Mukumba, P. Valorization of maize stover into biogas for heat and power generation: A South African perspective. *Fermentation* **11**(6), 338. 10.3390/fermentation11060338 (2025).

[30] Gesteiro, N. et al. Breeding dual-purpose maize: Grain production and biofuel conversion of the stover. *Agronomy* **13**(5), 1352. 10.3390/agronomy13051352 (2023).

[31] Jindo, K. et al. Assessment of trade-off balance of maize stover use for bioenergy and soil erosion mitigation in Western Kenya. *Front. Sustain. Food Syst.* **9**, 1409457. 10.3389/fsufs.2025.1409457 (2025).

[32] Allito, B. B. et al. Legume-Rhizobium specificity effect on nodulation, biomass production and partitioning of faba bean (Vicia faba L.). *Sci. Rep.* **11**, 3678. 10.1038/s41598-021-83235-8 (2021). 33574503 10.1038/s41598-021-83235-8 PMC7878908

[33] Serafin‑Andrzejewska, M., Falkiewicz, A., Wojciechowski, W. & Kozak, M. Yield and seed quality of faba bean (*Vicia faba* L. var. minor) as a result of symbiosis with nitrogen‑fixing bacteria. *Agriculture* **15**(9), 960. 10.3390/agriculture15090960 (2025).

[34] Denton, M. D., Pearce, D. J. & Peoples, M. B. Nitrogen contributions from faba bean (*Vicia faba* L.) reliant on soil rhizobia or inoculation. *Plant Soil* **365**(1), 363–374. 10.1007/s11104-012-1393-2 (2012).

[35] Gómez, L. D. et al. Valorising faba bean residual biomass: Effect of farming system and planting time on the potential for biofuel production. *Biomass Bioenerg.* **107**, 227–232. 10.1016/j.biombioe.2017.10.019 (2017).

[36] Jasinskas, A., et al. Corn, Hemp and Bean Multi-Crop Biomass Pellets and/or Fertilizer. Patent LT6998B. Available online: https://search.vpb.lt/pdb/patent/dossier/48325/text (2022).

[37] IUSS Working Group WRB. *World Reference Base for Soil Resources: International soil classification system for naming soils and creating legends for soil maps* 4th edn. (Austria, 2022).

[38] Romaneckas, K. et al. Short-term impact of multi-cropping on some soil physical properties and respiration. *Agron.* **12**, 141. 10.3390/agronomy12010141 (2022).

[39] Romaneckas, K., Švereikaitė, A., Kimbirauskienė, R., Sinkevičienė, A. & Balandaitė, J. The energy and environmental evaluation of maize, hemp and faba bean multi-crops. *Agronomy* **13**, 2316. 10.3390/agronomy13092316 (2023).

[40] Jolliffe, I. T. & Cadima, J. Principal component analysis: A review and recent developments. *Philos. Trans. R Soc. Math. Phys. Eng. Sci.* **374**, 1–16. 10.1098/rsta.2015.0202 (2016). 10.1098/rsta.2015.0202 PMC4792409 26953178

[41] Allego, D. L. & Gonzaga, A. B. Jr. Agronomic and carbon productivity of corn (Zea mays Linn.) and corn–legume intercropping under conservation agriculture practice system (CAPS). *IJRSI.* **11**(4), 1–12. 10.51244/IJRSI.2024.1104001 (2024).

[42] Wondire, Z., Tadele, Y. & Kechero, Y. Effects of maize–lablab intercropping on agronomic traits, forage quality, and economic viability. *Discov. Appl. Sci.* **7**(10), 1220 https://doi.org/10.1007/s42452-025-07805-5 (2025).

[43] Fisher, J., Bohm, H. & Heβ, J. Maize–bean intercropping yields in Northern Germany are comparable to those of pure silage maize. *Eur. J. Agron.* **112**, 125–147. 10.1016/j.eja.2019.125947 (2020).

[44] Hirpa, T. Effect of intercrop row arrangement on maize and haricot bean productivity and the residual soil. *GJSFR.* **14**, 1–9 (2014).

[45] Li, Z. et al. The synergistic priming effect of exogenous salicylic acid and H2O2 on chilling tolerance enhancement during maize (*Zea mays* L.) seed germination. *Front. Plant Sci.* **8**, 1153. 10.3389/fpls.2017.01153 (2017). 28725229 10.3389/fpls.2017.01153 PMC5496956

[46] Balandaitė, J. Crop biodiversity influence on multi-crops sustainability, productivity and energy efficiency. Doctoral Thesis. Vytautas Magnus University, Kaunas, Lithuania, (2023).

[47] Balandaitė, J., Romaneckas, K., Kimbirauskienė, R. & Sinkevičienė, A. Comprehensive assessment of the effect of multi-cropping on agroecosystems. *Plants* **13**(10), 1372. 10.3390/plants13101372 (2023). 10.3390/plants13101372 PMC11124805 38794442

[48] Li, Y. et al. Maize–soybean relay cropping increases soybean yield synergistically by extending the post-anthesis leaf stay-fresh period and accelerating grain filling. *Crop J.* **11**(6), 1921–1930. 10.1016/j.cj.2023.05.011 (2023).

[49] Yang, Z. et al. GmPTF1 modifies root architecture responses to phosphate starvation primarily through regulating GmEXPB2 expression in soybean. *Plant J.* **107**, 525–543. 10.1111/tpj.15307 (2021). 33960526 10.1111/tpj.15307

[50] Yang, H. et al. Yield photosynthesis and leaf anatomy of maize in inter- and mono-cropping systems at varying plant densities. *Crop J.* **10**, 893–903 (2022).

[51] Corre-Hellou, G., Fustec, J. & Crozat, Y. Interspecific competition for soil N and its interaction with N2 fixation, leaf expansion and crop growth in pea–barley intercrops. *Plant Soil* **282**, 95–208 (2006).

[52] Wu, Y. et al. Combining modelling and experiment to quantify light interception and inter row variability on intercropped soybean in strip intercropping. *Eur. J. Agron.* **164**, 127508. 10.1016/j.eja.2025.127508 (2025).

[53] Peñafiel–Sandova, Z. B. Effect of urea on lead absorption in corn (*Zea mays* L), spinach (*Spinacia olerácea* L.) and cabbage (*Brassica olerácea* L.). *Agron. Colomb.* **38**(2), 205–217. 10.15446/agron.colomb.v38n2.85082 (2021).

[54] Corre-Hellou, G., Hauggaard-Nielsen, H. & Jensen, E. S. Intercropping winter wheat with pea: Effects on growth, nitrogen uptake and chlorophyll content. *Field Crops Res.* **95**(2–3), 347–358. 10.1016/j.fcr.2005.04.007 (2006).

[55] Pierre, J. F. et al. Effect of maize–legume intercropping on maize physio-agronomic parameters and beneficial insect abundance. *Sustainability* **14**(19), 12385. 10.3390/su141912385 (2022).

[56] Stoltz, E. & Nadeau, E. Effects of intercropping on yield, weed incidence, forage quality and soil residual N in organically grown forage maize (*Zea mays* L.) and faba bean (*Vicia faba* L.). *Field Crops Res.* **169**, 21–29. 10.1016/j.fcr.2014.09.004 (2014).

[57] Shtaya, M. J. Y. et al. Effects of crop mixtures on rust development on faba bean grown in Mediterranean climates. *Crop Prot.* **146**, 105686. 10.1016/j.cropro.2021.105686 (2021).

[58] Streit, J., Meinen, C., Nelson, W. C. D. & Rauber, R. Above and belowground biomass in a mixed cropping system with eight novel winter faba bean genotypes and winter wheat using FTIR spectroscopy for root species discrimination. *Plant Soil* **36**, 141–158. 10.1007/s11104-018-03904-y (2019).

[59] Ciampitti, I. A. et al. Revisiting biological nitrogen fixation dynamics in soybeans. *Front. Plant Sci.* **12**, 727021. 10.3389/fpls.2021.727021 (2021). 34691106 10.3389/fpls.2021.727021 PMC8529188

[60] Nurgi, N., Tana, T., Dechassa, N., Tesso, B. & Alemayehu, Y. Effect of spatial arrangement of faba bean variety intercropping with maize on yield and yield components of the crops. *Heliyon* **9**(6), e16751. 10.1016/j.heliyon.2023.e16751 (2023). 37292354 10.1016/j.heliyon.2023.e16751 PMC10245057

[61] Dzvene, A. R., Tesfuhunei, W. A., Walker, S. & Ceronio, G. Optimizing the planting time and stand density of sunn hemp intercropping for biomass productivity and competitiveness in a maize-based system. *Field Crops Res.* **304**, 109179. 10.1016/j.fcr.2023.109179 (2023).

[62] Bybee-Finley, K. A., Mirsky, S. B. & Ryan, M. R. Crop Biomass not species richness drives weed suppression in warm-season annual grass–legume intercrops in the northeast. *Weed Sci.* **65**, 669–680 (2017).

[63] Balandaitė, J. et al. Impact of multi‑cropping on some soil physical properties, nutrient proportion and enzymatic activity. *Acta Agric. Scand. Sect. B Soil Plant Sci.*10.1080/09064710.2025.2590236 (2025).

[64] Branca, C., Blasi, C. D. & Galgano, A. Experimental analysis about the exploitation of industrial hemp (*Cannabis sativa*) in pyrolysis. *Fuel Process. Technol.* **162**, 20–29. 10.1016/j.fuproc.2017.03.028 (2017).

[65] Hu, E. et al. Pyrolysis behaviors of corn stover in new two-stage rotary kiln with baffle. *J. Anal. Appl. Pyrol.* **161**, 105398. 10.1016/j.jaap.2021.105398 (2022).

[66] Aubin, M. P. et al. Industrial hemp response to nitrogen, phosphorus, and potassium fertilization. *Crop Forage Turfgrass Manag.* **1**(1), 1–10. 10.2134/cftm2015.0159 (2016).

[67] Šarauskis, E. et al. Energy balance, costs and CO_2_ analysis of tillage technologies in maize cultivation. *Energy* **69**, 227–235. 10.1016/j.energy.2014.02.090 (2014).

[68] Petlickaitė, R. et al. Evaluation of the processing of multi-crop plants into pelletized biofuel and its use for energy conversion. *Processes* **11**(2), 421. 10.3390/pr11020421 (2023).

[69] Petlickaitė, R. et al. Evaluation of multi-crop biofuel pellet properties and the life cycle assessment. *Agriculture* **14**, 1162. 10.3390/agriculture14071162 (2024).

