# Supplementary material for: Biomass formation and yield performance in diverse multicrops and their potential for biofuel use in short-growing boreal climate conditions
Source: Sci Rep. 2026 Mar 30;16:10665. doi: 10.1038/s41598-026-46324-0 (PMC13040002; doi:10.1038/s41598-026-46324-0)
Supplement: Supplementary file 1 — Supplementary Material 1 [file 41598_2026_46324_MOESM1_ESM.docx]

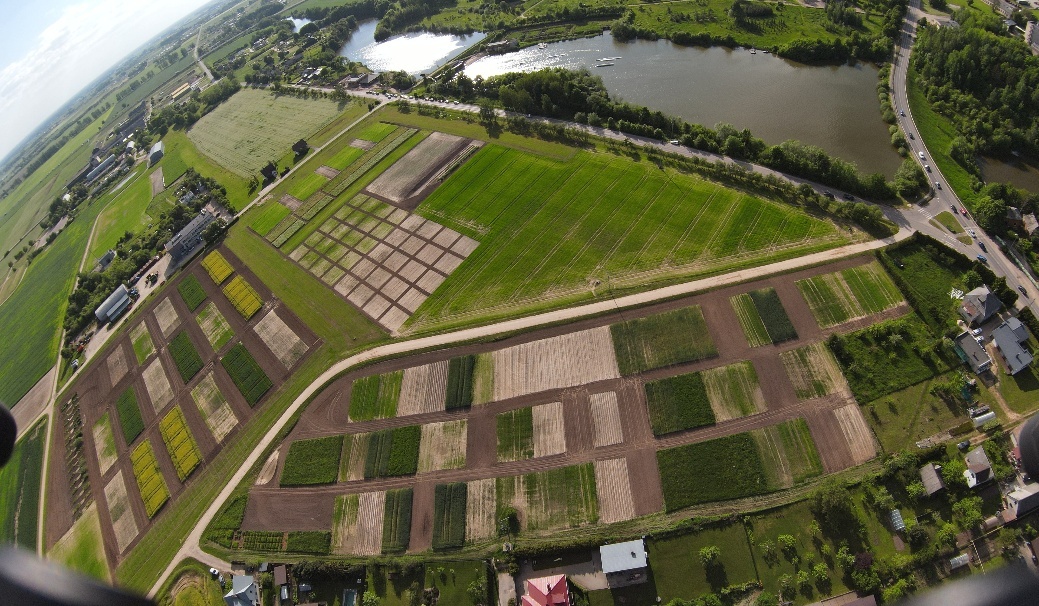


**Figure 1**. Fields of VMU AA Experimental Station.

a)

b)

**Figure 2.** Meteorological conditions during the experiment, Kaunas Meteorological Station.

| 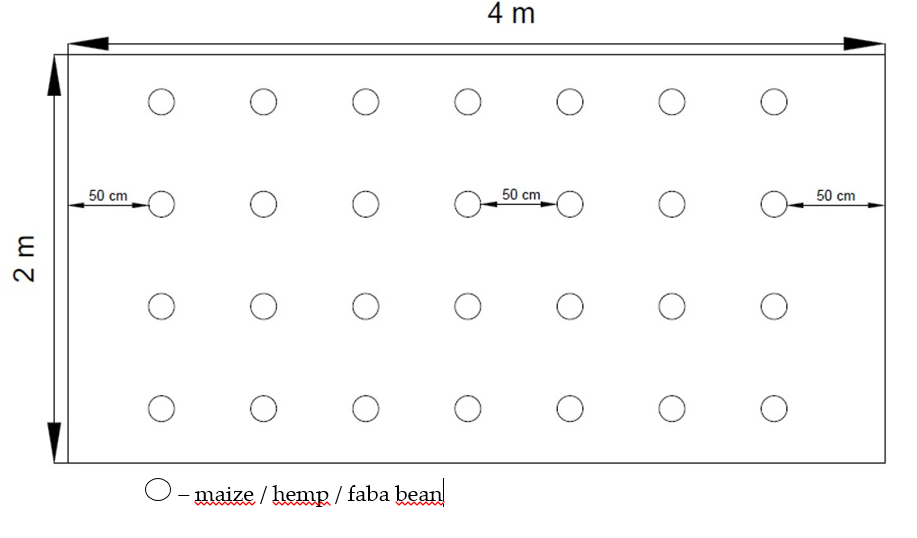 |
| --- |
| (a) |
| 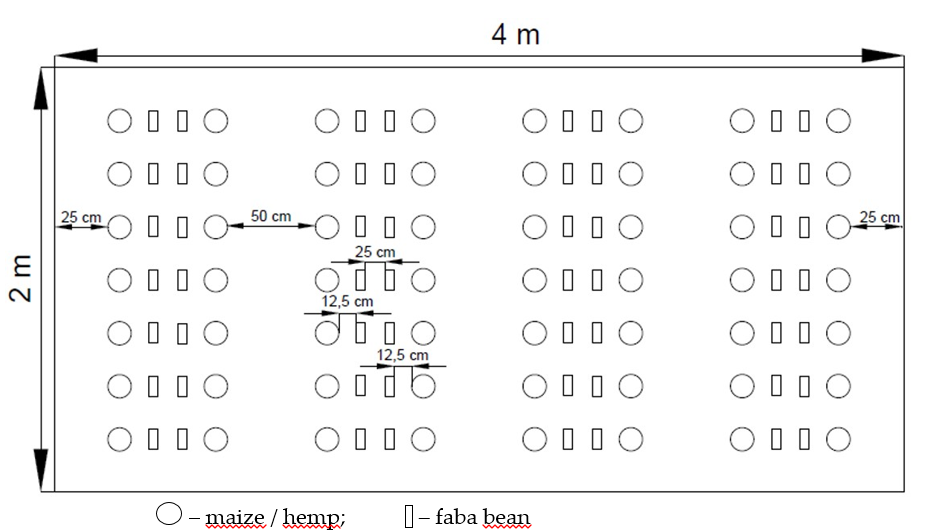  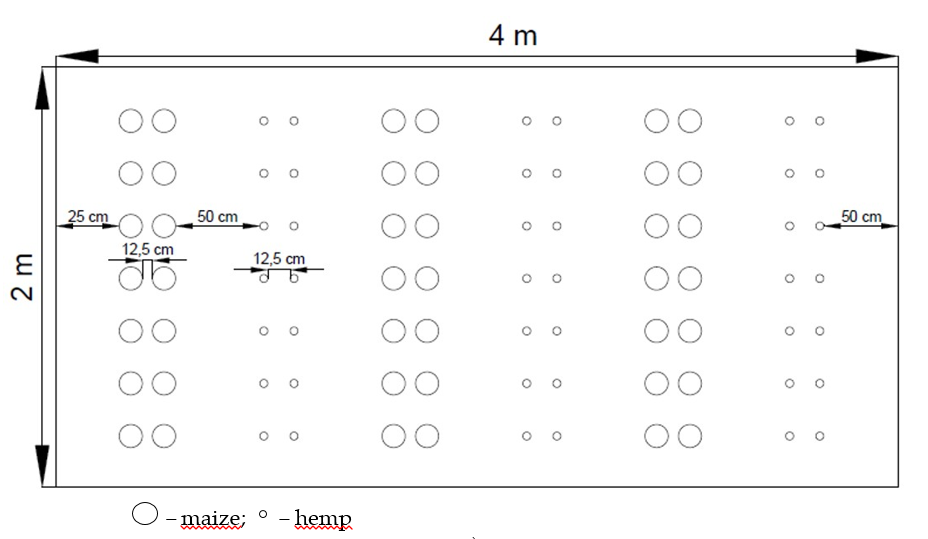 |
| (b) |
| 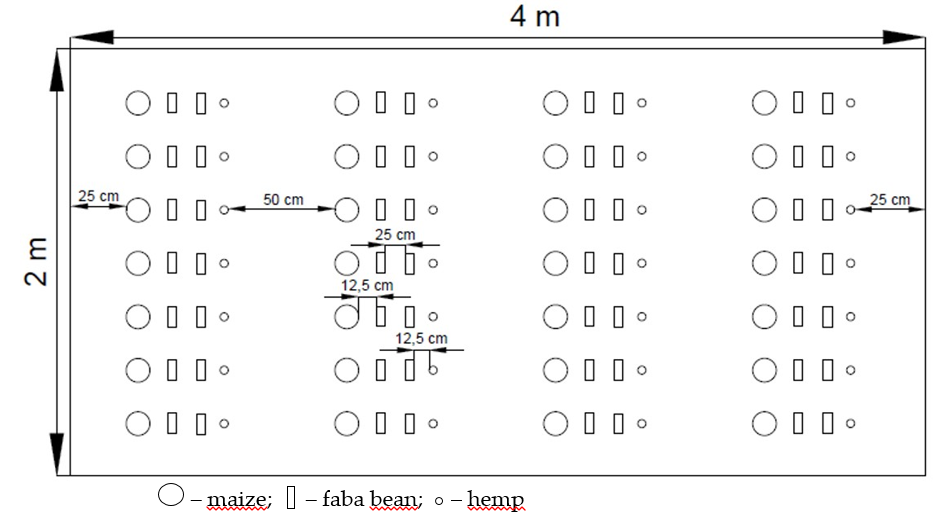 |
| (c) |

**Figure 3.** The sowing scheme of single (a), binary (b) and ternary (c) cultivations [38].


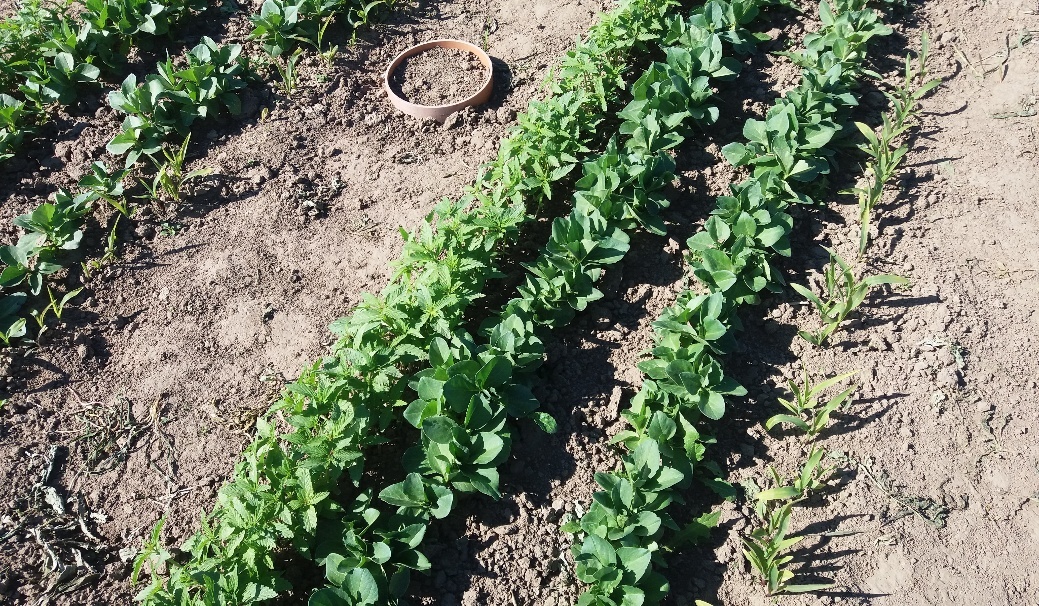

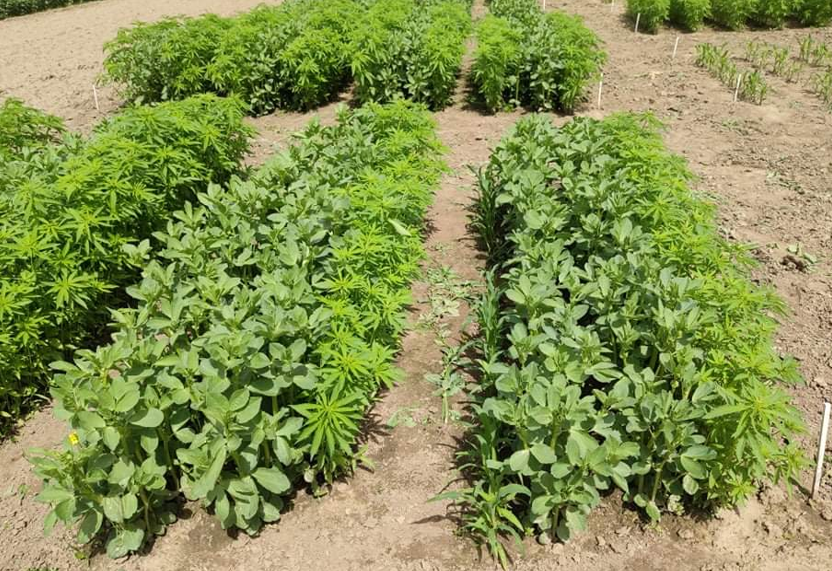


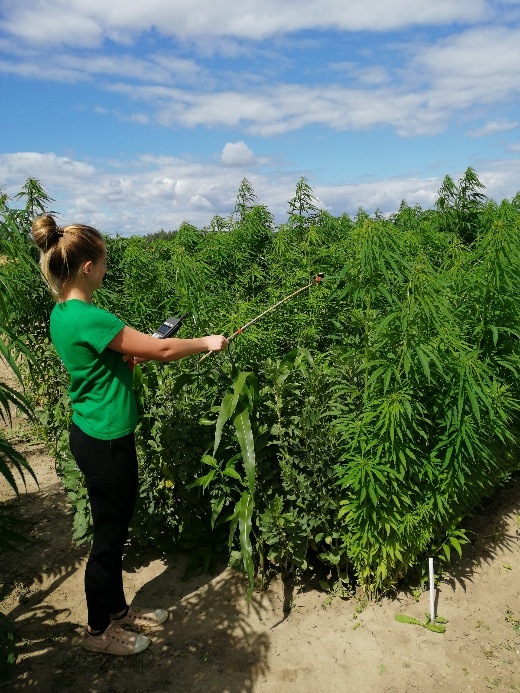


**Figure 4.** Development of ternary technical hemp, maize and faba bean crop.

| Crop diversification | | | |
| --- | --- | --- | --- |
| **M** | **M** + H | **M** + FB | **M** + H + FB |
| 127 | 138 | 181 | 73 |
| **H** | M + **H** | **H** + FB | M + **H** +FB |
| 140 | 93 | 131 | 86 |
| **FB** | M + **FB** | H + **FB** | M + H + **FB** |
| 65 | 71 | 66 | 67 |

Note: M – maize single crop, H – hemp single crop, FB – faba bean single crop, M+H – binary maize and hemp crop, M+FB – binary maize and faba bean crop, H+FB – binary hemp and faba bean crop, M+H+FB – ternary maize, hemp and faba bean crop.

**Table 1.** Seed rates, number m^-2^.

| **Crop operations** | **Execution time** | | |
| --- | --- | --- | --- |
|  | **2020** | **2021** | **2022** |
| Seedbed preparation | 22/04/2020 | 22/04/2021 | 18/04/2022 |
| Seeding | 30/04/2020 | 28/04/2021 | 22/04/2022 |
| Interrow tillage | 29/05/2020  15/06/2020 | 08/06/2021  25/07/2021 | 18/05/2022  08/06/2022 |
| Biomass harvest | 03/09/2020 | 24/08/2021 | 29/08/2022 |

**Table 2.** Crop operations.

| Biomass chemical composition | M | H | FB | M +H | M +FB | H + FB | M + H + FB |
| --- | --- | --- | --- | --- | --- | --- | --- |
| pH | 6.51 | 7.07 | 6.63 | 6.66 | 6.43 | 7.09 | 6.87 |
| Total nitrogen % | 0.92 | 0.64 | 2.12 | 0.84 | 1.48 | 1.22 | 0.98 |
| Available phosphorus % | 0.20 | 0.16 | 0.38 | 0.20 | 0.32 | 0.28 | 0.22 |
| Available potassium % | 1.20 | 0.76 | 0.78 | 0.98 | 1.04 | 0.98 | 0.87 |

Note: M – maize single crop, H – hemp single crop, FB – faba bean single crop, M+H – binary maize and hemp crop, M+FB – binary maize and faba bean crop, H+FB – binary hemp and faba bean crop, M+H+FB – ternary maize, hemp and faba bean crop.

**Table 3.** Biomass chemical composition.

| Parameter | M | H | FB | M+H | M+FB | H+FB | M+H+FB |
| --- | --- | --- | --- | --- | --- | --- | --- |
| Density, kg m^−3^ | 1077.67 ± 90.73 | 249.78 ± 80.08 | 1210.04 ± 109.72 | 1164.78 ± 159.60 | 1160.21 ± 39.95 | 1211.54 ± 77.51 | 1238.20 ± 104.57 |
| Ash content, % | 5.75 ± 0.07 | 8.57 ± 0.14 | 8.02 ± 0.07 | 6.87 ± 0.10 | 6.03 ± 0.14 | 7.12 ± 0.18 | 5.98 ± 0.10 |
| LCV, MJ kg^−1^ | 16.99 ± 0.34 | 16.73 ± 1.11 | 16.72 ± 0.18 | 16.87 ± 0.58 | 16.95 ± 0.41 | 16.81 ± 0.04 | 16.79 ± 0.76 |
| SST,° C | 948 ± 0.82 | 798 ± 1.42 | 810 ± 2.00 | 822 ± 0.34 | 923 ± 0.77 | 723 ± 0.20 | 1042 ± 0.14 |

Note: M – maize single crop, H – hemp single crop, FB – faba bean single crop, M+H – binary maize and hemp crop, M+FB – binary maize and faba bean crop, H+FB – binary hemp and faba bean crop, M+H+FB – ternary maize, hemp and faba bean crop. LCW—lower calorific value, SST—ash shrinkage starting temperature.

**Table 4.** Main characteristics of multi crop solid fuel pellets (according to Petlickaitė et al., [68]).

Note: M–maize single crop, H–hemp single crop, FB–faba bean single crop, M+H–binary maize and hemp crop, M+FB–binary maize and faba bean crop, H+FB–binary hemp and faba bean crop, M+H+FB–ternary maize, hemp and faba bean crop. CEI–complex evaluation index, * – average of evaluation points (EPs), ** – standard deviation of EPs, *** – standard deviation of the average of the evaluation points below the evaluation threshold.

**Figure 5.** Comprehensive assessment of crop diversification in terms of crop total dried biomass (according to Balandaitė et al., [47]).

| Treatments | Diesel fuel consumption L ha^−1^ | Energy input  MJ ha^−1^ | Energy output  MJ ha^−1^ | Net energy  MJ ha^−1^ |
| --- | --- | --- | --- | --- |
| M | 70.7 | 10178.7 | 78959.7 | 68781.0 |
| H | 79.0 | 10176.6 | 150030.8 | 139854.2 |
| FB | 70.7 | 16745.5 | 166787.0 | 150041.5 |
| M+H | 84.8 | 11511.0 | 205080.5 | 193569.5 |
| M+FB | 76.5 | 19348.6 | 134842.3 | 115493.7 |
| H+FB | 84.8 | 18266.6 | 183074.0 | 164807.4 |
| M+H+FB | 103.3 | 20162.9 | 387831.0 | 367668.1 |

Note: M–maize single crop, H–hemp single crop, FB–faba bean single crop, M+H–binary maize and hemp crop, M+FB–binary maize and faba bean crop, H+FB–binary hemp and faba bean crop, M+H+FB–ternary maize, hemp and faba bean crop.

**Table 5.** Fuel consumption and energy indices of cropping systems (according to the Romaneckas et al., [39]).
